# Graphene-Based 3D Scaffolds in Bone Regeneration: Emerging Opportunities for MRONJ Treatment

**DOI:** 10.3390/pharmaceutics18030335

**Published:** 2026-03-08

**Authors:** Claudio Catalano, Giulia Gerini, Gian Marco Podda, Valentina Palmieri, Massimiliano Papi, Giordano Perini, Antonio Minopoli, Marco De Spirito, Gianluca Tenore, Simona Ceccarelli, Guido Caldarelli, Umberto Romeo

**Affiliations:** 1 Department of Oral and Maxillofacial Sciences, Sapienza University, 00161 Rome, Italy; catalano.1972389@studenti.uniroma1.it (C.C.); gianmarco.podda@uniroma1.it (G.M.P.); gianluca.tenore@uniroma1.it (G.T.); umberto.romeo@uniroma1.it (U.R.); 2 Department of Experimental Medicine, Sapienza University, 00161 Rome, Italy; giulia.gerini@uniroma1.it (G.G.); simona.ceccarelli@uniroma1.it (S.C.); 3 Institute for Complex Systems, National Research Council, 00185 Rome, Italy; guido.caldarelli@cnr.it; 4 Department of Neuroscience, Catholic University of Sacred Heart, Largo Francesco Vito 1, 00168 Rome, Italy; massimiliano.papi@unicatt.it (M.P.); giordano.perini@unicatt.it (G.P.); antonio.minopoli@unicatt.it (A.M.); marco.despirito@unicatt.it (M.D.S.)

**Keywords:** graphene, materials, osteonecrosis, scaffold, regeneration, MRONJ

## Abstract

**Background**: Medication-related osteonecrosis of the jaw (MRONJ) remains a challenging complication associated with antiresorptive and antiangiogenic therapies, characterized by impaired bone healing, infection, and compromised vascularization. Advanced biomaterials capable of promoting bone regeneration and modulating the local microenvironment are being investigated as potential therapeutic strategies. Graphene-based biomaterials have recently emerged as promising candidates due to their unique physicochemical properties and multifunctional biological effects. **Objective**: This systematic review aimed to analyze and synthesize current evidence on graphene-based 3D scaffolds and related graphene-based biomaterials for bone regeneration, with particular attention to their potential relevance in MRONJ treatment and prevention. **Data Sources**: A systematic literature search was conducted in PubMed and Scopus databases, complemented by manual screening of reference lists from relevant publications. **Eligibility Criteria**: Studies investigating graphene-based scaffolds, composites, or graphene-derived biomaterials for bone regeneration were considered. Experimental in vitro and in vivo studies, as well as translational studies addressing osteogenesis, angiogenesis, antimicrobial activity, immunomodulation, or drug-delivery properties relevant to bone healing and MRONJ, were included. Editorials, conference abstracts, and non-English publications were excluded. **Methods**: Titles and abstracts were independently screened by the authors, followed by full-text assessment for eligibility. Data regarding scaffold composition, graphene derivatives, biological mechanisms, and regenerative outcomes were qualitatively synthesized due to heterogeneity in study designs and outcome measures. **Results**: The identified literature highlights the multifaceted role of graphene-based biomaterials in bone regeneration. Graphene and its derivatives enhance osteogenic differentiation, promote angiogenesis, modulate immune responses, and exhibit intrinsic antimicrobial properties. In addition, graphene-based scaffolds provide versatile platforms for drug delivery and photothermal or photodynamic therapeutic strategies. These multifunctional properties may address key pathophysiological mechanisms involved in MRONJ, including impaired bone remodeling, infection control, and tissue regeneration. **Limitations**: The available evidence is predominantly derived from preclinical studies, with limited direct investigation in MRONJ-specific models and considerable heterogeneity in scaffold design and experimental methodologies. **Conclusions**: Graphene-based 3D scaffolds represent a promising and versatile platform for bone regenerative strategies potentially applicable to MRONJ management. Further translational research and well-designed preclinical and clinical studies are required to clarify their safety, efficacy, and therapeutic applicability. **Registration**: This review was conducted according to PRISMA 2020 guidelines. The review protocol was not registered.

## 1. Introduction

Medication-related osteonecrosis of the jaw (MRONJ) is a rare but clinically significant complication observed in patients undergoing treatment with anti-resorptive or anti-angiogenic agents. From an epidemiological standpoint, the incidence of MRONJ varies considerably depending on the patient population and the type of pharmacological treatment administered [1,2]. In oncology patients receiving high-dose therapy for bone metastases or multiple myeloma, the risk of developing MRONJ increases substantially, reaching between 1% and over 10%, depending on the duration of therapy and the presence of additional risk factors [3,4,5].

Initially described as bisphosphonate-related osteonecrosis of the jaw (BRONJ), the condition has expanded in both its epidemiological and clinical scope with the introduction of newer therapeutic agents, such as denosumab and various angiogenesis inhibitors, necessitating a redefinition of the terminology and diagnostic criteria [6]. For the purposes of this manuscript, the designation BRONJ will be employed solely to denote the subset of MRONJ cases arising specifically from bisphosphonate therapy.

Bisphosphonates (BPs) are composed of two phosphonate chains attached to a central carbon atom, which is, in turn, bonded to two side chains, R1 and R2 [6]. The R1 side chain is responsible for the drug’s affinity for hydroxyapatite, the mineral component of bone, while the R2 side chain determines the drug’s potency. Based on the presence or absence of an amino group in the R2 side chain, BPs are classified into two pharmacological categories: aminobisphosphonates (NBPs)—including zoledronate, pamidronate, alendronate, risedronate, ibandronate, and neridronate—and non-aminobisphosphonates, such as clodronate, tiludronate, and etidronate. NBPs exhibit greater affinity for bone and possess a potency 10 to 1000 times higher than that of BPs lacking amino groups. To date, NBPs represent the principal class of BPs associated with the development of BRONJ [7,8,9]. In oncology patients without bone metastases, NBPs are used for the prevention or treatment of drug-induced osteoporosis, such as that occurring following hormone therapy for breast cancer (pre- and postmenopausal) or prostate cancer [3,10,11]. The other drug causing MRONJ, denosumab, is a monoclonal antibody that acts by forming immune complexes with Receptor Activator of Nuclear Factor kB Ligand (RANKL), thereby inhibiting the recruitment and activation of osteoclasts in a temporary and reversible manner, resulting in a reduction in bone turnover [12,13]. Denosumab clinical indications are based on the treatment of bone metastases and giant cell tumors of the bone. Also, denosumab has been recommended for the treatment of osteoporosis, to increase bone mass in women receiving adjuvant hormone therapy for non-metastatic breast cancer, and in men undergoing hormone therapy for prostate cancer [10].

MRONJ often manifests following invasive dental procedures, such as tooth extractions, and is characterized by exposed or probable necrotic bone persisting over eight weeks, accompanied by symptoms like pain, swelling, fistula formation, and secondary infection [14].

The pathogenesis of MRONJ is multifactorial, involving a convergence of suppressed bone remodeling, compromised vascular supply, cellular toxicity, and microbial infection. BP accumulation and inhibition of osteoclast-mediated resorption occur through interference with the mevalonate pathway, a fundamental cascade for the synthesis of isoprenoids, leading to osteoclast apoptosis and long-term remodeling suppression. This effect predisposes bone to necrosis when local repair is required. Denosumab produces a similar osteoclast-inhibitory effect through RANKL blockade. Moreover, anti-angiogenic drugs may further hinder healing by impairing neovascularization. It has been demonstrated that the two pharmacologic agents (BPs and denosumab) that potently target/inhibit osteoclast function through diverse mechanisms result in indistinguishable clinical and experimental disease phenotypes; however, denosumab brings a higher incidence of MRONJ, likely due to the more robust resorption inhibition [15].

Local oral conditions significantly modulate MRONJ risk. Persistent dentoalveolar infections, such as periodontal or periapical disease, promote inflammation, alter local pH, and create bacterial niches that foster necrosis. Invasive dental procedures, chiefly tooth extraction, are reported in over half of MRONJ cases and serve as critical triggers in the presence of underlying compromised bone turnover. The mandible is disproportionately affected due to its comparatively limited blood supply [12]. Systemic factors such as diabetes mellitus, concurrent chemotherapy, corticosteroid therapy, and cancer metastases intensify vulnerability by further impairing tissue repair and immune function. Lifestyle and oral hygiene factors—poor plaque control, removable prostheses irritation, and tobacco use—contribute additional risk by maintaining chronic inflammatory stimuli at the bone interface [16].

In summary, MRONJ arises from an interplay of targeted pharmacodynamics—osteoclast inhibition and angiogenesis suppression—combined with local oral pathology, invasive dental events, and patient-specific systemic conditions. This complex, multifactorial etiology necessitates a multidisciplinary prevention strategy.

Currently available therapeutic approaches for MRONJ primarily rely on conservative management strategies, including antimicrobial mouth rinses, systemic antibiotic therapy, and pain control, as well as surgical interventions ranging from sequestrectomy to marginal or segmental resection in advanced stages. Adjunctive regenerative treatments such as leukocyte–platelet-rich fibrin, ozone therapy, hyperbaric oxygen therapy, and low-level laser therapy have been proposed to enhance wound healing and angiogenesis. However, these approaches largely address secondary clinical manifestations, such as infection and inflammation, rather than the underlying pathophysiological mechanisms of MRONJ, including impaired bone remodeling, reduced vascularization, and immune dysregulation. As a result, clinical outcomes remain variable and frequently require extensive surgical procedures that may further compromise already ischemic bone tissue.

Given the frequent association with dental procedures, composites enriched with nanomaterials might be designed to be used locally at extraction sockets or surgical sites to provide both structural support and biological cues, thereby reducing the risk of necrotic progression while simultaneously promoting bone healing. In this context, multifunctional scaffolds incorporating graphene-based materials have demonstrated the ability to regulate stem cell differentiation, macrophage polarization, and pro-angiogenic signaling pathways in a dose- and surface chemistry-dependent manner. Graphene is indeed a two-dimensional carbon material composed of a single layer of sp^2^-hybridized atoms arranged in a hexagonal lattice, and it has been extensively studied in dentistry over the past decade, with research highlighting its mechanical strength, antibacterial activity, biocompatibility, and potential to enhance osseointegration and bone regeneration in dental implants and restorative materials [17,18]. Its unique honeycomb structure gives graphene and its derivatives exceptional mechanical strength (Young’s modulus around 1 TPa, and tensile strength up to 130 GPa), along with excellent electronic and photocatalytic properties, making them highly promising for biomedical applications. In particular, graphene-based materials (GBMs) have been shown to enhance osteogenic differentiation while also improving mechanical performance when incorporated into nanocomposites [19]. This combination of bone-like strength and biological activity makes GBMs particularly exciting candidates for bone tissue engineering.

Furthermore, the integration of graphene-based scaffolds with near-infrared (NIR)-mediated phototherapy may provide additional therapeutic advantages by enabling light-triggered antibacterial activity, modulation of reactive oxygen species levels, and enhancement of vascular and osteogenic responses. This combinatorial approach has the potential to simultaneously address multiple components of MRONJ pathogenesis, including infection control, impaired angiogenesis, and defective bone healing, thereby representing a promising strategy for improving clinical outcomes in patients at risk of post-extraction osteonecrosis.

The aim of this systematic review was to identify and synthesize the available evidence on graphene-based 3D scaffolds and related graphene-based biomaterials for bone regeneration, with specific focus on mechanisms and translational relevance for MRONJ prevention and treatment.

## 2. Search Strategy

**Scheme 1:**
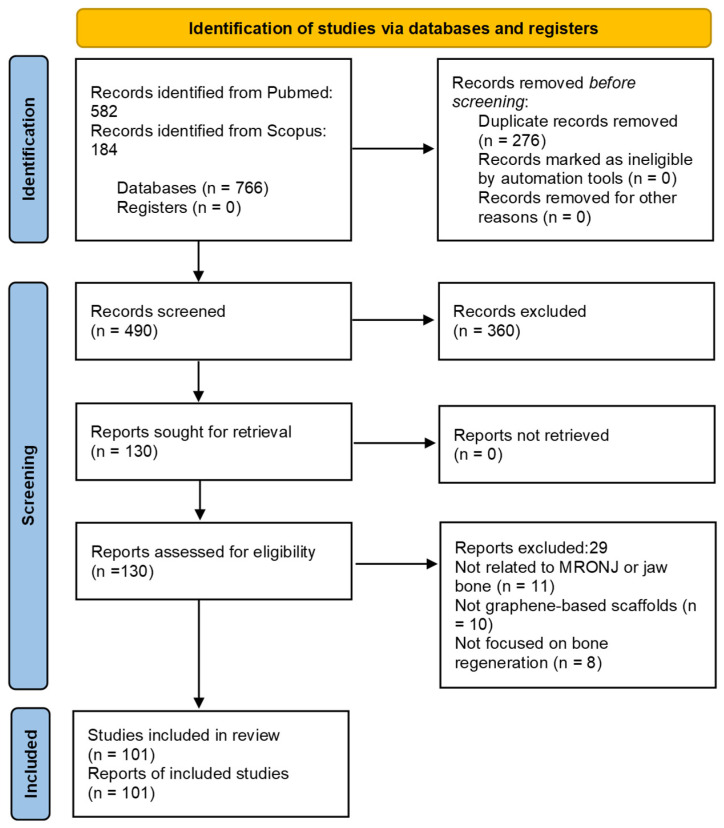
Preferred Reporting Items for Systematic Reviews and Meta-Analyses (PRISMA) 2020 flow diagram for the systematic review.

This review was conducted in accordance with the Preferred Reporting Items for Systematic Reviews and Meta-Analyses (Scheme 1) guidelines to ensure a transparent and reproducible literature selection process, checklist is reported in Supplementary Material File S1 [20]. A systematic literature search was performed across the PubMed and Scopus databases to identify relevant studies investigating the application of GBMs for bone regeneration, with particular emphasis on their potential relevance to MRONJ. The search included publications from January 2015 to December 2025. The search strategy combined controlled vocabulary and free-text terms using Boolean operators as follows: (“graphene*” OR “graphene oxide” OR “GO” OR “reduced graphene oxide” OR “rGO”) AND (“bone regeneration” OR “osteogenesis” OR “scaffold*” OR “3D print*” OR “tissue engineering”) AND (“MRONJ” OR “osteonecrosis of the jaw” OR “BRONJ” OR “jaw bone”). Inclusion criteria comprised peer-reviewed original articles and review studies published in English that investigated graphene-based three-dimensional scaffolds or composite biomaterials for bone regeneration in in vitro, in vivo, or translational contexts potentially relevant to MRONJ pathophysiology. Editorials, conference abstracts, letters to the editor, and studies unrelated to bone tissue engineering were excluded. Following database retrieval, duplicate records were removed. Titles and abstracts were independently screened for relevance by the authors, and potentially eligible studies were subsequently assessed through full-text evaluation. Reference lists of the articles included were manually screened to identify additional relevant studies.

## 3. Physiological Processes and Mechanisms of Tissue Regeneration Following Tooth Extraction

MRONJ typically presents after invasive dental interventions, most commonly tooth extractions [1,9,21]. The proposed etiopathogenetic hypotheses relate to the traumatic action exerted during the surgical procedure on bone with altered metabolism caused by the anti-resorptive and/or anti-angiogenic activity of drugs, which alter and delay the normal healing processes.

**Figure 1:**
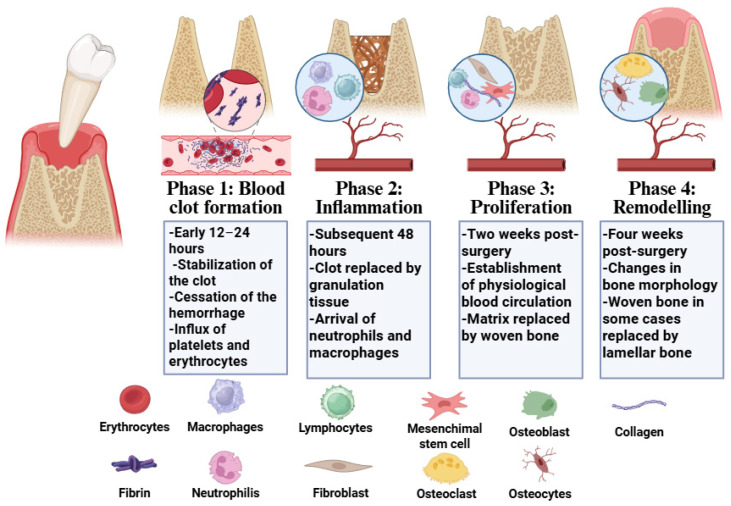
**Biological phases of post-extraction socket healing.** Healing after tooth extraction progresses through four sequential and partially overlapping stages. In the first phase, hemostasis and coagulation occur within the initial 24 h and are characterized by the formation of a blood clot composed of red and white blood cells, platelets, and fibrin. The inflammatory stage begins after 24 h and extends through the first week, with infiltration of neutrophils, macrophages, and lymphocytes that progressively replace the clot with granulation tissue. From the second week, the proliferation stage is initiated by fibroplasia and revascularization, leading to the formation of a provisional matrix that is gradually substituted by woven bone. Finally, from the fourth week, woven bone undergoes qualitative changes and is ultimately replaced by lamellar bone and bone marrow. Created in BioRender. Papi, M. (2026) https://BioRender.com/2cxgoq2.

Tooth extraction triggers a complex healing process involving morphological changes and loss of ridge height, width, and volume, with high individual variability [21]. The healing sequence consists of four overlapping phases and follows intramembranous ossification, with no cartilage stage, as shown in Figure 1 [22,23,24].

In the hemostasis and coagulation phase, a clot forms in the socket. Hemostasis begins to control bleeding through three coordinated phases: (i) primary hemostasis: vascular spasm and vasoconstriction occur, and platelets adhere to exposed subendothelial matrix via von Willebrand Factor activate, degranulate, aggregate, and form a platelet plug; (ii) secondary hemostasis: coagulation cascades convert fibrinogen to fibrin, stabilizing the clot; and (iii) tertiary hemostasis: plasmin dissolves the fibrin clot to restore blood flow [25]. The thrombus acts as a temporary matrix for inflammatory cell migration and releases cytokines and growth factors (as interleukin-1 β (IL-1β), Platelet-Derived Growth Factor (PDGF), Transforming Growth Factor-β (TGF-β), Fibroblast Growth Factor (FGF), Vascular-Endothelial Growth Factor (VEGF), and Insulin-like Growth Factor (IGF)), initiating the inflammatory and proliferative phases of healing.

The inflammatory phase begins within 24–72 h post-extraction, activating the innate immune system in a microbe-rich oral environment. Injured cells release calcium waves, reactive oxygen species (ROS), lipid mediators, and chemokines, triggering polymorphonuclear neutrophils (PMNs) to swarm the site [26]. Platelet degranulation releases cytokines and chemokines to attract more immune cells. Monocytes arrive and differentiate into M1 macrophages that produce matrix metalloproteases (MMPs), clear pathogens, and perform neutrophil efferocytosis. Failure to clear neutrophils prolongs inflammation. Resolution involves a switch to M2 macrophages, which mimic endothelial cells, promote angiogenesis, release anti-inflammatory mediators (e.g., interleukin-10, IL-10), and stimulate fibroblast proliferation, extracellular matrix (ECM) synthesis, and osteoblast activation. Granulation tissue forms from macrophages, fibroblasts, and vascular sprouts. By 1–4 weeks, the initial clot transforms into granulation tissue in a centrifugal pattern, enabling transition to the proliferative phase once the site is cleared and stabilized. Between weeks 2 and 8 post-extraction, in the proliferative phase, granulation tissue is replaced by a fibrovascular provisional matrix and woven bone. Fibroplasia involves fibroblast proliferation and ECM deposition (collagen, elastin, and proteoglycans), and is driven by TGF-β, Fibroblast Growth Factor 2 (FGF-2), and PDGF. The matrix—rich in mesenchymal cells, vessels, and collagen—supports migration and adhesion. High expression of collagen I (bone marker) and MMPs (ECM remodeling, angiogenesis) is observed. Over 50% of matrix cells show osteogenic potential (Runx2, osteocalcin). Angiogenesis occurs simultaneously.

Local levels of VEGF increase markedly [27] and disruptions in VEGF signaling have been shown to impair socket healing [28], underscoring the essential role of local VEGF in normal tissue repair. Endothelial cells, stimulated by VEGF and FGF, form new vessels through basal lamina breakdown and cell migration. Multiple lines of evidence indicate that any disturbance of VEGF delays socket healing and induces the onset of MRONJ. Indeed, VEGF primarily originates from osteoblasts and is bound to the bone matrix: Deleting *Vegfa* in osteoblast lineage cells greatly reduces VEGF production at bone repair sites [29]. VEGF has been detected in osteoblasts, not osteocytes. Specifically, VEGF from early osteolineage cells (but not mature osteoblasts or osteocytes) plays a key role in angiogenesis and bone formation during fracture repair [30].

Woven bone appears as early as 2–4 weeks: Bone forms as mesenchymal cells differentiate under TGF-β and bone morphogenetic protein-7 (BMP-7) influence. Bone morphogenetic proteins (BMPs) also promote growth factor release (VEGF, FGF, PDGF, and IGF). Osteogenic cells originate from bone marrow, periodontal ligament (PDL), periosteum, adipocytes, and pericytes [31].

PDL fibers disappear by 2–4 weeks (though some persist longer), and residual tissues (e.g., epithelial rests, cementicles) are replaced. Osteoclasts contribute to bundle bone remodeling, while bone-forming cells and vessels invade the matrix, generating primary osteons and sometimes parallel-fibered bone. Both processes are regulated by osteoblasts/osteoclasts via signaling pathways (RANK/RANKL/OPG, RUNX2, M-CSF, OC, OPN, and OST) [31,32]. Woven bone, initially weak, will later remodel into lamellar bone and marrow. Epithelial tissue proliferates centrally over the matrix, progressively sealing the socket [24].

## 4. Biological Challenges in MRONJ Tissue Regeneration

Although MRONJ has a multifactorial etiology, three mechanistic models can be proposed: (i) **Model 1**: Bone resorption and angiogenesis are independently disrupted, both contributing to MRONJ; (ii) **Model 2**: Inhibition of angiogenesis leads to reduced bone resorption, resulting in MRONJ; and (iii) **Model 3**: Suppression of bone resorption causes impaired angiogenesis, ultimately leading to MRONJ.

**Model 1** can be excluded due to the significant evidence linking bone resorption and angiogenesis. For example, treatment with zoledronic acid reduces circulating angiogenic factors (VEGF, PDGF, and TGF-β), while animal studies show decreased neovascularization in BRONJ models. Denosumab and its analog OsteoProteGerin Factor (OPG-Fc) have demonstrated anti-angiogenic effects in cancer patients and mice, respectively. Inhibiting the interaction between RANKL and its receptor, denosumab blocks osteoclast maturation and survival, thereby reducing osteoclast-mediated bone resorption [33]. Angiogenic factors such as VEGF, PDGF, and FGF-2 also directly promote osteoclast differentiation and activity. Inhibition or genetic deletion of VEGF suppresses bone resorption, indicating a reciprocal relationship [34].

**Model 2**, where inhibition of angiogenesis reduces bone resorption, thereby leading to MRONJ, is also unlikely. Anti-angiogenic drugs like sunitinib and sorafenib reduce bone turnover markers modestly, far less than BPs at osteoporotic doses, and such moderate reductions in resorption are insufficient to explain ONJ cases linked solely to anti-angiogenic drugs [35,36]. Osteocytes are damaged by BPs and contribute significantly to MRONJ [37]. Furthermore, combining anti-resorptive and anti-angiogenic agents increases MRONJ risk but does not significantly lower bone resorption markers compared to anti-resorptives alone. BPs like zoledronic acid reduce circulating VEGF levels up to 21 days after treatment [38] and accumulate mainly in the bone matrix, where they remain active even when plasma levels drop sharply. This suggests that the drug’s impact on VEGF reduction is mediated by inhibition of matrix-bound VEGF release.

Increasing evidence supports **Model 3**, in which suppression of bone resorption causes impaired angiogenesis, ultimately leading to MRONJ. Anti-resorptive agents significantly decrease both circulating angiogenic factors and local vascularity. Although bevacizumab more effectively reduces circulating VEGF, it has little effect on other angiogenic pathways. By contrast, zoledronic acid reduces VEGF, PDGF, and FGF-2 simultaneously. BPs and denosumab impair angiogenesis in extraction sockets and reduce vascular structures in periodontal tissues during MRONJ progression. Notably, MRONJ incidence is lower in patients on anti-angiogenics compared to those on anti-resorptives [6]. Finally, the synergy between anti-resorptive and anti-angiogenic agents in improving cancer outcomes likely stems from their shared suppression of angiogenesis. BPs inhibit prenylation of GTPases, disrupting the cytoskeleton and resorption activity. However, osteocyte death—not just osteoclast apoptosis—is central to the pathogenesis of MRONJ, as shown by necrosis and reduced turnover in BP-treated jawbones [39]. Despite their apoptotic effect, BPs may paradoxically increase the number and size of osteoclasts over long-term treatment, as a compensatory response [40], possibly aided by their stimulation of osteoblast proliferation [41]. The RANK/RANKL pathway, mediated by osteocytes and osteoblasts, plays a crucial role in osteoclast activation. Taken together, the data support a model in which anti-resorptive therapy leads to local angiogenic suppression, contributing to ischemic bone necrosis.

**Figure 2:**
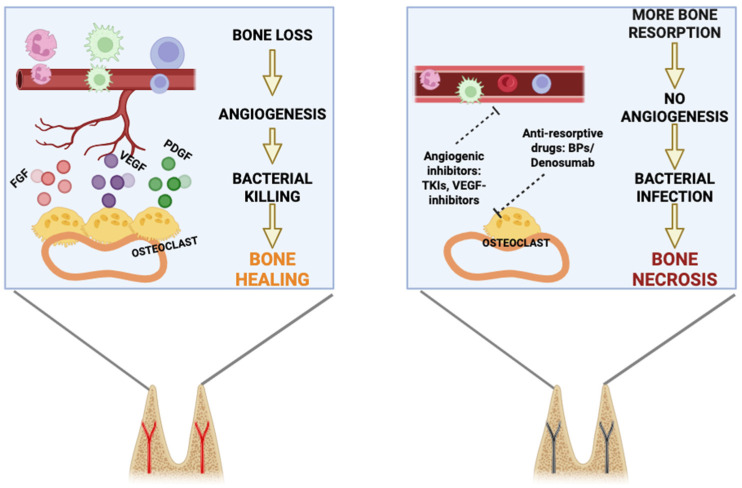
**Schematic comparison between bone healing, bone necrosis, and bacterial infection.** Panel on the left illustrates physiological bone healing, where osteoclast-mediated bone resorption promotes the release of matrix-embedded growth factors. Panel on the right depicts the pathological process of bone necrosis induced by anti-resorptive agents. In this context, osteoclast activity and angiogenesis are suppressed, preventing the release of growth factors, impairing vascularization, and establishing a vicious cycle of reduced bone resorption. Created in BioRender. Papi, M. (2026) https://BioRender.com/2cxgoq2.

Another hypothesis, alternative or coexisting, concerns pre-existing infectious issues (i.e., chronic inflammatory dento-periodontal and peri-implant diseases). Indeed, numerous studies have highlighted early signs and symptoms of MRONJ clinically associated with chronic infectious–inflammatory dento-periodontal processes prior to tooth extraction [42]. In other words, rather than the extraction procedures themselves, chronic infectious–inflammatory dento-periodontal processes—especially those with a chronic course—are at higher risk of MRONJ onset. Exposed bone within an extraction socket becomes particularly susceptible to bacterial infection in patients receiving anti-resorptive or anti-angiogenic therapies. This increased vulnerability is largely due to the critical role of local vascularization in clearing bacteria after tooth extraction. This includes the release of pro-inflammatory cytokines, such as interleukin-1 (IL-1), interleukin-6 (IL-6), and TNF-α [43], that promote vasodilation and increase vascular permeability, facilitating the migration of immune cells from the bloodstream to the site of injury. When local blood flow is impaired, the recruitment of immune cells to the affected area is hindered, compromising bacterial clearance. In addition to reducing immune cell infiltration, impaired vascularization results in local hypoxia. This low-oxygen environment favors the growth of anaerobic bacteria, the majority of microbial species identified at MRONJ sites (Figure 2) [44]. Moreover, hypoxia negatively affects immune function: It reduces immune cell survival, diminishes phagocytic capacity, and lowers the production of ROS—all critical for effective pathogen elimination [45]. A schematic depiction of MRONJ occurrence is reported in Figure 2 modified from [46].

## 5. Clinical Treatment of MRONJ

MRONJ represents a clinically heterogeneous condition in which therapeutic decision-making must be guided not only by disease stage but also by the nature of the triggering event. The surgery-triggered form, associated with invasive dentoalveolar procedures (reported in proportions ranging from 45–61% to 62–82%), and a non-surgery-triggered form, arising in the absence of recent surgical intervention, typically linked to chronic inflammatory or mechanical insults, can occur [6,47,48]. The surgery-triggered form accounts for the majority of documented cases, with tooth extraction representing the most frequent precipitating event [6,47,48]. The non-surgery-triggered form constitutes a smaller proportion (often estimated at 20–40%, with truly spontaneous or chronic-factor cases reaching up to 35–50% in specific studies) [48,49].

Although both entities share the same pharmacological substrate, their biological onset, temporal progression, and clinical behavior differ substantially. Consequently, treatment strategies must be tailored to optimize healing outcomes while minimizing the risk of disease progression or recurrence.

### 5.1. Surgery-Triggered MRONJ

Management of this surgery-triggered MRONJ initially relies on conservative therapy aimed at minimizing additional surgical insult while attempting to restore a biologically favorable healing environment [6]. First-line treatment in early clinical stages includes antimicrobial mouth rinses (0.12–0.2% chlorhexidine), systemic antibiotic therapy during inflammatory exacerbations (e.g., amoxicillin/clavulanic acid combined with metronidazole for 7–14 days), analgesic support when necessary, and strict oral hygiene reinforcement combined with periodic clinical monitoring [50,51]. However, once necrotic bone sequestration or persistent infection occurs, surgical intervention becomes unavoidable. In such cases, the therapeutic objective is the complete removal of non-vital bone tissue up to bleeding margins, followed by tension-free primary mucosal closure. Depending on lesion extension, surgical management may range from superficial osteoplasty and dentoalveolar curettage to sequestrectomy and marginal resection, while segmental resection with immediate reconstruction using titanium plates or vascularized flaps may be required in advanced stages [52]. Preventive strategies such as immediate alveolar ridge preservation (ARP) performed after extraction using xenogeneic, allogenic, or synthetic biomaterials with or without barrier membranes have been reported to reduce ridge resorption by approximately 1–2.5 mm compared with spontaneous healing, particularly in the coronal buccal region of the ridge [52,53].

### 5.2. Non-Surgery-Triggered MRONJ

Non-surgery-triggered MRONJ is commonly associated with persistent dento-periodontal infection, peri-implantitis, or chronic mucosal trauma caused by ill-fitting removable prostheses [48,49].

Treatment is primarily focused on the elimination of infectious foci, stabilization of periodontal or peri-implant conditions, reduction in mechanical irritation, and prevention of mucosal breakdown. Conservative endodontic therapy and non-surgical periodontal treatment are therefore preferred whenever feasible in order to preserve natural dentition and avoid extractions that may precipitate secondary necrosis [48,49]. Pharmacological management remains similar to that adopted for surgery-triggered MRONJ; however, temporary suspension of anti-resorptive therapy (“drug holiday”), when clinically permissible, may exert greater therapeutic benefit in selected non-BRONJ cases, particularly those associated with denosumab due to its reversible mechanism of action. Conversely, in bisphosphonate-related MRONJ, the long-term skeletal accumulation of the drug limits the clinical impact of temporary discontinuation [54]. Surgical treatment in these patients is generally reserved for lesions refractory to conservative management and is performed using minimally invasive techniques in order to avoid further disruption of vascularized bone tissue.

### 5.3. Stage-Adapted Multimodal Treatment

Regardless of the triggering mechanism, MRONJ management ultimately relies on a stage-adapted multimodal strategy integrating infection control, pain management, and surgical removal of necrotic tissue when indicated. Minor surgical procedures such as superficial osteoplasty, dentoalveolar curettage, or sequestrectomy are typically employed in focal lesions, whereas marginal or segmental resections are reserved for diffuse or complicated stages based on clinical and radiological evaluation using computed tomography or magnetic resonance imaging [54].

Adjunctive biostimulatory therapies—including leukocyte–platelet-rich fibrin (L-PRF), ozone therapy, hyperbaric oxygen therapy (20–40 sessions), and photobiomodulation by low-level laser therapy (LLLT; 808–910 nm)—may be employed as supportive treatments to enhance mucosal healing, stimulate angiogenesis, and reduce inflammatory burden [55,56,57]. LLLT has been shown to promote fibroblast proliferation, collagen deposition, osteoblastic differentiation, and local microcirculation, resulting in reduced pain, edema, bone exposure, and fistula formation in selected clinical reports (see also Section 6.3).

Nevertheless, medium- to advanced-stage surgical protocols are not devoid of significant risks. Highly demolitive interventions may further compromise already ischemic bone tissue and represent an additional surgery-triggered risk factor for MRONJ recurrence, particularly in medically compromised patients. Notably, several of the currently adopted adjunctive protocols already employ laser systems operating within the near-infrared (NIR) range (808–900 nm), which overlaps with the irradiation window suitable for graphene-based photothermal activation, thereby suggesting a potential synergistic translational advantage for scaffold-assisted regenerative approaches.

## 6. Graphene-Based Biomaterials for Osteonecrosis Treatment

Given the high incidence of MRONJ following tooth extraction, a rational design of biomaterials combined with the local controlled release of bioactive factors or antimicrobial agents, potentially supported by laser therapy, may significantly reduce its occurrence [58].

Several biomaterials for promoting bone healing have been developed in the form of filling sponges, membranes, hydrogels, nanofibers, particles, and powders [22,59,60]. Biomaterials can be employed for their intrinsic functionality and/or as carriers for delivering bioactive substances and living cells, thereby further promoting healing. Among biomaterials, GBMs have been extensively investigated in the field of regenerative medicine, owing to their ability to modulate cellular behavior in a highly controlled manner. As described above, graphene is endowed with exceptional electronic, mechanical, and chemical properties that have spurred intense research in recent years. Its derivative, Graphene oxide (GO), is a chemically modified form characterized by the presence of oxygen-containing functional groups such as epoxides, hydroxyls, and carboxyls on its surface, which profoundly influences its hydrophilicity, reactivity, and biological interactions. It is important to point out that surface chemical features make the behavior of GBMs highly tunable in biological environments, directly affecting their interactions with proteins, cell membranes, and intracellular signaling pathways [61,62]. As a result, GBMs can exert either pro-angiogenic effects, promoting blood vessel formation, or anti-angiogenic effects, inhibiting neovascularization, depending on factors such as concentration, particle size, and surface modifications.

GBMs can be successfully embedded in 3D printing filaments, enabling the fabrication of customized scaffolds and dental implants with enhanced functional properties. The incorporation of GBM improves mechanical strength, electrical conductivity, and bioactivity while producing patient-specific scaffolds that not only support tissue regeneration but also promote cellular responses, making them highly promising for applications in implantology and bone tissue engineering [63,64]. In the following section, we will review studies reporting how the biological effects of GBMs could potentially be harnessed for the management of MRONJ, although specific investigations directly addressing the use of graphene in this pathology remain scarce.

### 6.1. Graphene-Activated Biological Pathways Relevant in Bone Regeneration

**Figure 3:**
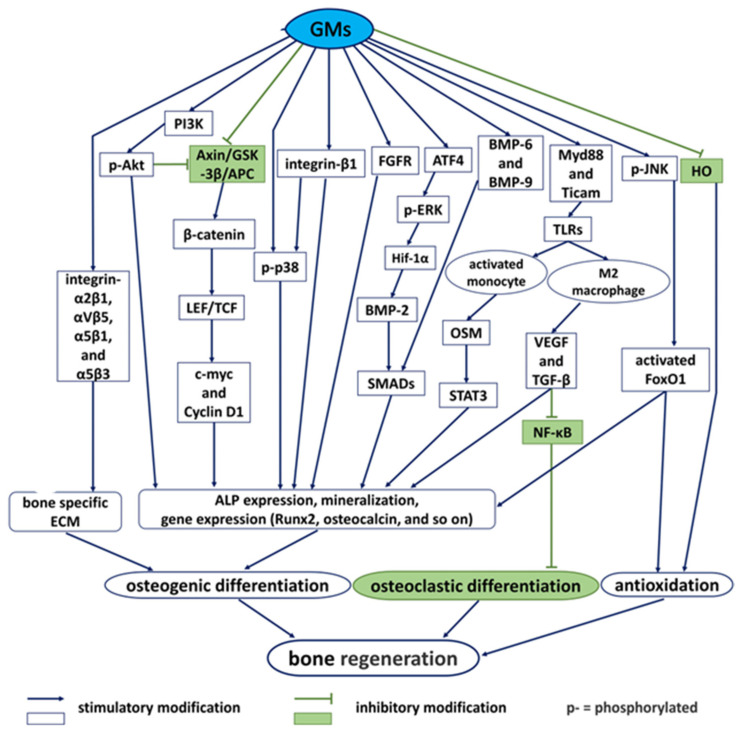
**Osteogenic signaling pathways influenced by GBMs**. Modified with permission from [19] © 2021 John Wiley & Sons Ltd.

Adult mesenchymal stem cells derived from dental tissues have strong self-renewal and differentiation abilities, enabling regeneration of dental and various other tissues and making them highly promising for regenerative medicine and dentistry [65]. In the last 15 years of research on the properties of GBMs, it has been extensively ascertained that graphene has intrinsic osteogenic differentiation properties in the absence of any chemical inducer, that is, spontaneous osteogenic differentiation in stem cells. Effects are resumed in Figure 3.

Due to mechanical and surface properties, graphene upregulates gene and protein expressions of Runx2 and OCN, while odontoblast cell-related genes (Msx1, PAX-6, and DMP-1) were significantly downregulated in dental pulp stem cells (DPSCs) [66]. In addition to affecting signaling molecules, the anchor points provided by graphene wrinkles on the surface increase the collagen production [67] and orient matrix deposition [68]. Stiff graphene surface acts indeed as physical stimulation to initiate a mechanotransduction-related signaling cascade of osteogenic signals [69], activating the integrin-FAK transmembrane complex, which, in turn, activates ROCK1 and F-actin. Then, the phosphorylation of SMAD p1/5 is stimulated, and the expression of Runx2, OPN, and OCN is promoted. In the study of Dubey et al. [67], it has been demonstrated that the expressions of talin, FAK, vinculin, and β-integrin in graphene-coated samples are significantly higher than in controls. Similar to graphene, GO can also spontaneously induce osteogenic differentiation of mesenchymal stem cells (MSCs). Due to the existence of high-density oxygen in GO, the GO substrate has hydrophilicity and high adsorption properties for proteins and soluble growth factors, making it capable of interacting with cells [70]. GO-mediated spontaneous osteogenic differentiation is also attributed to the physical stimulation generated by its rough and wrinkled surface to MSCs, similarly to graphene. This effect is enhanced with the increase in GO layer number [71]. GO also enhances spontaneous osteogenesis by affecting Fas [72] through regulation of FAK/P38 signaling pathways [73]. Rosa et al. [74] demonstrated that GO not only increased the expression of osteogenic genes of DPSCs but also promoted the expression of DMP-1 and DSPP, which are closely related to odontogenic differentiation of DPSCs.

The chemical nature of oxygen-containing functional groups on the surface of GO can be modulated to influence MSC differentiation. Tiberio et al. demonstrated that reducing these oxygen groups in rGO results in a more negatively charged surface, which enhances hydroxyapatite (HA) deposition and alters the associated protein adsorption profile. This biologically active apatite layer, in turn, promotes the differentiation of mesenchymal stem cells [75]. Furthermore, rGO-based substrate enhances calcium deposition in the ECM produced by osteoblast-like cells with an increased expression of the osteogenic markers mandatory for mineralization, which benefits from the ability of GBMs to adsorb osteogenic factors [76].

The canonical Wnt/β-catenin signaling is one of the main osteogenic pathways activated by GBMs, which relies on the cytoplasmic stabilization of β-catenin, enhancing osteoblastic differentiation and bone regeneration [77,78,79].

As previously pointed out, in the process of bone healing and osteogenesis, the immune system and the bone system always interact with each other. The existing research results indicate that interleukin-4 (IL-4), IL-10, IL-13, TGF-β, and Oncostatin M (OSM) can promote osteogenesis, while IL-1 and IL-6 have the opposite effect [80,81,82,83]. The effects of TNF-α on osteogenesis are contradictory [84] and probably related to the dose [85].

GBMs are known to regulate M1-M2 macrophage polarization, particularly the M2 type, limiting inflammatory response and participating in tissue remodeling and repair. Studies indicated that the M2 type differentiation of macrophages is conducive to the differentiation of osteoblasts [86,87]. The presence of M2-type macrophages can promote the secretion of potential osteogenic factors such as VEGF, BMP-2, and TGF-β. It has been demonstrated that GO-based nanocomposites could regulate macrophage polarization by activating specific signaling pathways, such as TGF-β/BMP2 and VEGF, which promoted the repair of osteoporotic fractures [88].

Furthermore, the collective effects of M2 cytokines on different cell types may lead to increased calcium deposition and bone mineralization [83]. Some GBMs have been proven to induce macrophages to differentiate into M2 type. Nishida et al. showed that GO application significantly increased osteoblastic MC3T3-E1 cell proliferation, thus promoting bone induction in dog tooth extraction sockets. Similarly, Zou et al. corroborated that the GO-carboxymethyl chitosan hydrogel loaded with IL-4 and BMP-2 can induce macrophage differentiation into M2-type and enhance the ability of bone marrow mesenchymal stem cells (BMSCs) with osteogenic differentiation in vitro [89]. GO could regulate macrophage polarization through Toll-like receptors (TLRs) [90]. On the other hand, M2 cytokines can inhibit osteoclastic differentiation by downregulating the nuclear factor kappa-B (NF-κB) pathway [83,91]. Indeed, Xiong et al. proclaimed that a reduced GO/Zinc Silicate/Calcium Silicate electroconductive biocomposite could suppress osteoclastic differentiation of mouse leukemic monocyte macrophages (RAW 264.7 cells) induced by the RANKL [92]. Analogously, magnesium-enriched GO nanoscrolls promoted the regeneration of bone defects in rats through downregulating the NF-κB pathway [93]. A study indicated that the immune microenvironment induced by GO stimulates osteogenic differentiation of BMSCs through the OSM pathway [94]. As for OSM downstream signals, OSM can regulate the osteogenic differentiation of DPSCs by activating STAT3, and increase the expression of osteogenic differentiation markers, such as ALP and Runx2 [95,96].

Finally, different types of BMP have been proven to play a key role in the osteogenic differentiation of MSCs induced by GBMs, which promotes the secretion of BMP or can be loaded with BMP and used as a drug delivery system [97,98,99,100,101,102].

### 6.2. GBMs in Dentistry: Scaffold That Supports Bone Healing and Drug Delivery of Pro-Angiogenic Factors

The intrinsic pro-osteogenic and mechanical properties of GBMs have opened the research field of dentistry to composite materials designed for several applications, including bone tissue engineering, dental pulp and periodontal tissue regeneration, adhesives, cements, resin reinforcement, and coatings for dental implants and abutments [17].

GO has been incorporated into conventional dental materials such as resin-based composites, silane primers, and adhesives to improve their mechanical performance, as summarized in recent reviews [17,18]. Its application has also extended to coatings for dental and orthopedic implants, typically deposited by electrophoretic or physical methods, as well as to scaffolds for bone tissue engineering. GO has been combined with various biopolymers, including collagen, chitosan, gelatin, and alginate. For example, adding GO to collagen scaffolds enhanced compressive strength, enzyme resistance, calcium, and protein adsorption, and significantly promoted MC3T3-E1 osteoblast proliferation. In vivo, scaffolds containing 1 μg/mL GO stimulated cell and tissue ingrowth and angiogenesis in rat subcutaneous tissue, and achieved nearly fivefold higher bone formation in dog extraction sockets compared with collagen alone. Overall, GO enhances the mechanical robustness of biopolymer-based scaffolds, expands structural design possibilities, and has also been successfully integrated with ceramics such as HA and calcium phosphate (CaP) to further support bone tissue engineering applications [103].

In addition to promoting bone differentiation, graphene-based materials are also known to interfere with the production of pro-angiogenic factors. VEGF gene expression after endothelial progenitor cell (EPC) exposure to different rGOs is modulated by their surface chemistry, suggesting a chemistry-dependent effect. Precisely, Polo-Montalvo et al. demonstrated that while soluble GO significantly decreased EPC viability, increased intracellular ROS production, and downregulated VEGFR2 expression, its thermally reduced counterparts (rGO15 and rGO30) mitigated these adverse effects. Remarkably, rGO30 not only preserved cell viability but also induced higher VEGFR2 expression than control cultures after 72 h, suggesting an early pro-angiogenic potential linked to its improved electroconductivity and surface characteristics [104].

The role of graphene, combined with chitosan, as a biological modulator has also been studied by Zhang et al., as they demonstrated that the generation of controlled levels of ROS contributed to the activation of Hypoxia-Inducible Factor 1-Alpha (HIF-1α), which binds to hypoxia response elements (HREs) in the VEGF promoter, thus increasing its transcription. In addition, in this study, they observed that moderate concentrations of GO (0.1–0.5 wt.%) increased the expression of several factors, such as Stromal-Derived Factor 1 (SDF-1), MMP-9, and the progenitor cell marker CD34, which acted synergistically to promote angiogenesis. Indeed, GO concentrations represent a limiting factor in developing such combined hydrogel scaffolds, since an excessive dose of GO (1.0 wt.%) has been reported to lead to toxicity, oxidative stress, and reduced angiogenic effects [105].

**Figure 4:**
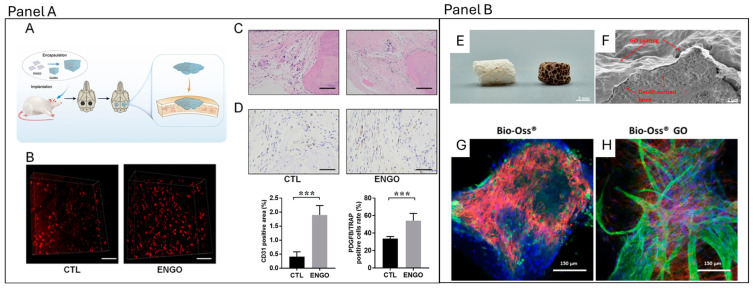
Panel (**A**): ENGO promotes preosteoclast-derived PDGFB and angiogenesis in a calvarial defect rat model. (**A**) Schematic of the ENGO/GelMA scaffold that was implanted into the calvarial defect model. (**B**) 3D images of administered rhodamine B showing angiogenesis in the rat calvarial defect area in different rat groups. Scale bars, 100 μm. (**C**) HE staining of tissues, scale bars 100 μm. (**D**) CD31 immunohistochemical staining of the defective region in the control and ENGO treatment groups. Scale bars, 50 μm. *** *p* < 0.001. Reproduced with permission from [108] (2024) American Chemical Society. Panel (**B**): Graphene Oxide-Coated Bio-Oss^®^. (**E**) Photograph of uncoated (left) and GO-coated (right) Bio-Oss^®^ scaffold. (**F**) SEM micrograph of broken GO-coated Bio-Oss^®^ scaffold with highlighted GO layer. Morphological appearance of vascular structures on GO-coated Bio-Oss^®^ (**G**,**H**) MSC/OEC co-cultures on Bio-Oss^®^ (**G**) and GO-coated Bio-Oss^®^ scaffolds (**H**) on day 7, visualized by confocal laser scanning microscopy. Cells were stained with VE-Cadherin (green), Phalloidin-TRITC (red), and Höechst (blue). Modified with permission from [106] © 2022 under the terms and conditions of the Creative Commons Attribution (CC BY) license.

Concerning graphene-based scaffolds, one of the most relevant findings is their ability to interact directly with VEGF at the material interface. The incorporation of GO within scaffold matrices not only promoted VEGF secretion by mesenchymal stem cells and endothelial cells but also facilitated VEGF binding to the scaffold surface, thereby prolonging its local availability. This retention effect enhanced the stability and bioactivity of VEGF in the peri-implant environment, ensuring sustained stimulation of endothelial cells and supporting the formation of organized vascular networks. The bound VEGF acted synergistically with the secreted fraction, amplifying pro-angiogenic signaling and contributing to a microenvironment highly conducive to neovascularization. In vivo, this translated into greater vessel density and accelerated integration of the scaffold with host tissue [106], as in Figure 4E–H.

Other studies indicate that graphene oxide/biphasic calcium phosphate (GO/BCP) composite scaffold significantly promotes the expression of the angiogenic genes (VEGF and ANG-1) in rat bone marrow mesenchymal stem cells (rBMSCs) [107].

The apparently contrasting findings highlight the remarkable plasticity of graphene-based materials, both (i) when employed in solution or incorporated into scaffolds, and (ii) depending on the specific chemical groups present on their surface.

Recently, it has been demonstrated that the PDGF pathway can also be activated by graphene through an electrochemically derived nanographene oxide (ENGO) hydrogel. In a rat calvarial defect model, ENGO treatment markedly enhanced angiogenesis with an increase in CD31 endothelial marker and increased platelet-derived growth factor-B (PDGF-B) expression in preosteoclasts compared with controls (Figure 4A–D from [108]). In vitro experiments further confirmed that ENGO suppressed RANKL-induced osteoclast differentiation, reducing the expression of osteoclast-associated markers such as OC-STAMP and cathepsin K while inhibiting bone resorption activity. At the same time, ENGO preserved and amplified the secretion of the osteoclast-derived coupling factor PDGF-BB, thereby promoting endothelial cell migration and tube formation. Mechanistically, ENGO downregulated isocitrate dehydrogenase 1 (IDH1), which, in turn, inhibited histone lysine demethylase 7A (KDM7A) activity, leading to an accumulation of the repressive histone mark H3K9me2 at the cathepsin K promoter. This epigenetic mechanism blocked osteoclast maturation while sustaining PDGF-BB release, ultimately creating a microenvironment favorable to angiogenesis and bone regeneration [108].

Incorporating graphene into scaffolds markedly increases their surface area and provides abundant binding sites for biomolecules, thereby enhancing the potential for drug loading and sustained release. This high surface-to-volume ratio enables the combination of graphene-based composites with pro-angiogenic factors, creating multifunctional scaffolds that simultaneously support osteogenesis and angiogenesis. Such hybrid systems have been shown to improve vascular ingrowth and bone regeneration compared with conventional biomaterials.

The study conducted by Ibrahim and colleagues evaluated a composite hydrogel composed of chitosan/polyvinyl alcohol (PVA) loaded with GO and titanium dioxide nanoparticles (nano TiO_2_) as a scaffold for the reconstruction of cortical bone defects in a canine model. The addition of GO significantly promoted and improved local angiogenesis, as evidenced by histological evaluation reporting abundant neovascularization and active cellular infiltration around the lesion site. It also facilitated the migration and proliferation of osteogenic cells, as revealed by immunohistochemistry with a significant increase in VEGF and osteogenic markers. Hydrogels with GO and TiO_2_ created a favorable environment for bone regeneration, thus showing improved defect closure [109].

In another study, VEGF was loaded into nanographene-coated internal fixation screws to enable a sustained release of bioactive VEGF for up to nine days without burst release. Li et al. demonstrated in a femoral neck fracture model that VEGF-loaded screws significantly enhanced angiogenesis and osteogenesis compared with controls, as evidenced by increased microvessel density, higher bone mineral density, greater new bone formation, and faster fracture healing [110].

Also, graphene-based scaffolds can be functionalized to deliver microRNAs (miRNAs), offering a powerful strategy for osteonecrosis treatment. In a recent study, an injectable alginate/HA hydrogel loaded with graphene oxide–polyethylenimine complexes carrying miR-7b (GPC@miR) was applied in rat models of femoral head osteonecrosis. The system provided controlled release of the miRNA while maintaining high transfection efficiency and low cytotoxicity. Mechanistically, GPC@miR inhibited the formation of bone-associated osteoclasts (BAOs) by downregulating DC-STAMP, thereby limiting bone resorption, while preserving and expanding vessel-associated osteoclasts (VAOs). These VAOs secreted pro-angiogenic and osteogenic factors, particularly VEGF-A and PDGF-BB, which drove type H vessel angiogenesis and coupled osteogenesis. In vivo, hydrogel injection restored VAO/BAO balance, enhanced vascularization, and promoted bone regeneration, ultimately delaying femoral head collapse in osteonecrosis models. This highlights graphene-based miRNA delivery systems as a minimally invasive therapeutic approach capable of reprogramming the bone microenvironment through simultaneous suppression of pathological resorption and stimulation of angiogenesis-driven bone repair [111,112].

GO has been proposed as a carrier for the local delivery of zoledronic acid, allowing effective bone-targeted activity at reduced drug concentrations. In a study by Boran et al., zoledronic acid was non-covalently conjugated to GO, achieving efficient drug loading through π–π interactions and hydrogen bonding. The optimized ZOL–GO complex, obtained using 50 µM zoledronic acid combined with 2.91 ng/mL GO, preserved cell viability of BM-MSCs while significantly enhancing mineralization compared with zoledronic acid alone. Notably, ZOL–GO complexes induced greater osteogenic mineral deposition at lower or intermediate zoledronic acid concentrations, whereas higher doses of free zoledronic acid (200 µM) impaired mineralization. These findings indicate that GO-based delivery systems can reduce the effective dose of zoledronic acid required to achieve osteogenic effects, potentially limiting dose-dependent adverse events associated with systemic bisphosphonate administration [113]. GO can also be incorporated into composite bone substitutes to improve both physicochemical and biological performance. Demir-Oguz et al. developed injectable calcium phosphate-based bone substitutes incorporating graphene oxide and zoledronic acid, demonstrating that GO significantly enhanced the mechanical properties of the composites while enabling controlled local release of zoledronic acid. In particular, GO incorporation increased compressive strength and promoted osteoblast proliferation, differentiation, and mineralization, while simultaneously exerting inhibitory effects on cancer cells in vitro [114]. A similar approach has been explored with alendronate, another widely used bisphosphonate. Zeng et al. developed graphene oxide-functionalized collagen sponges loaded with alendronate, achieving sustained local drug release for up to 30 days while preserving biocompatibility. The GO–alendronate composites promoted osteogenic differentiation of bone marrow mesenchymal stem cells and significantly inhibited osteoclastogenesis both in vitro and in osteoporotic rat models. Notably, local delivery via GO-based scaffolds enhanced bone regeneration and reduced osteoclast activity without the need for high systemic drug exposure, further supporting the potential of graphene oxide as a multifunctional carrier for bisphosphonates in bone regeneration strategies [115].

### 6.3. Photodynamic Therapy and Photobiomodulation in MRONJ Context

As previously explained, light-mediated therapies can improve tissue regeneration in MRONJ. Particularly, photobiomodulation (PBM) involves the application of low-intensity red or NIR light to stimulate cellular activity due to the absorption of photons by mitochondrial chromophores, particularly cytochrome c oxidase, leading to cellular responses that promote tissue repair, reduce inflammation, and alleviate pain.

Moreover, photodynamic therapy (PDT) emerged as a minimally invasive adjunct therapy to combat biofilm-associated infections and antibiotic resistance. Several clinical trials have validated its efficacy across various dental applications, including the treatment of dental caries and periodontic infections, denture stomatitis, peri-implantitis, and endodontic infections [116].

**Figure 5:**
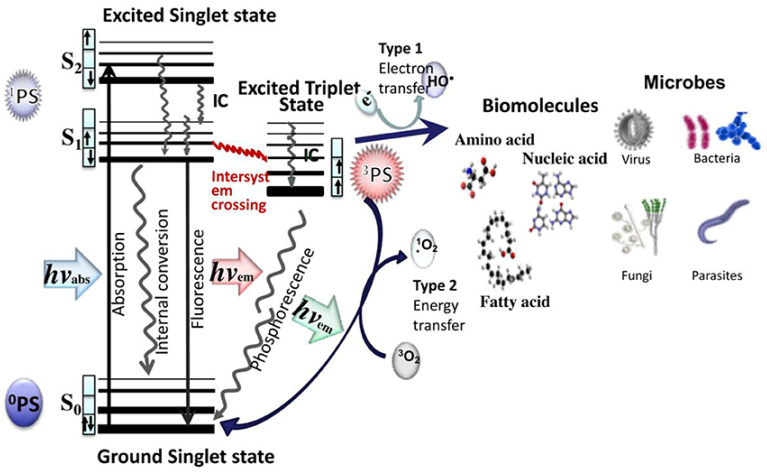
**Jablonski diagram depicting the photophysical and photochemical processes of a photosensitizer (PS) during photodynamic action.** After absorption of light (hν), the PS is excited from the ground singlet state (S_0_) to higher singlet states, relaxes to the first excited singlet state (S_1_), and can then undergo internal conversion, fluorescence emission, or intersystem crossing to reach the excited triplet state (T_1_). From that triplet state, the PS engages in Type I (electron or proton transfer to biomolecules generating reactive radicals) or Type II (energy transfer to molecular oxygen to form singlet oxygen, ^1^O_2_) reactions, which lead to damage of biomolecules and microbial cells. Reproduced with permission from [117] under Creative Commons Attribution License (CC BY).

PBM and PDT share the use of specific light wavelengths but differ significantly in their mechanisms of action, clinical objectives, and biological effects. PBM uses non-ionizing light sources primarily to promote tissue repair. PBM modulates oxidative stress by balancing ROS: At low levels, these molecules act as beneficial signals that stimulate cellular proliferation, while excessive oxidative damage is attenuated. Another key factor is the release of nitric oxide, which induces vasodilation and improves local microcirculation. PBM also influences the inflammatory response, downregulating pro-inflammatory mediators while upregulating anti-inflammatory signals, thus creating a microenvironment more favorable to healing. PBM stimulates MSCs, enhancing the production of IL-10 and, most importantly, VEGF. NIR irradiation at 1064 nm has been shown to preserve MSC viability and to promote tissue healing by reducing inflammation and fostering the formation of new blood vessels. PDT combines a light-sensitive molecule or photosensitizer(PS) with light exposure at a specific wavelength to generate ROS, which selectively induce cytotoxic effects in targeted cells. After the photosensitizer is administered and accumulates in diseased tissues, light activation leads to oxidative damage, causing apoptosis or necrosis. Upon light absorption, the PS moves from a ground singlet state to an excited singlet state and, through intersystem crossing, to a long-lived triplet state. From this triplet state, two primary mechanisms can occur (Figure 5):

- Type I reactions involve electron transfer from the excited PS to nearby molecules, resulting in the formation of superoxide anion (O_2_•^−^), hydrogen peroxide (H_2_O_2_), and hydroxyl radicals (HO•).
- Type II reactions involve direct energy transfer to molecular oxygen, producing singlet oxygen (^1^O_2_), a highly cytotoxic species.

Both pathways lead to oxidative stress and subsequent damage to cellular components, including membranes, proteins, and DNA. Importantly, singlet oxygen and hydroxyl radicals are the most reactive species but have the shortest diffusion distances, enabling localized and selective cytotoxicity. Furthermore, under specific conditions, Type III mechanisms involving oxygen-independent pathways have also been proposed [116].

No established gold standards currently exist for NIR light therapy parameters in scaffold-assisted MRONJ treatment, as available studies report heterogeneous protocols without consensus, particularly with regard to stage-specific or site-tailored irradiation strategies. PBM based on red and near-infrared wavelengths has been shown to act at the mitochondrial level through activation of cytochrome c oxidase, leading to enhanced ATP production, controlled release of reactive oxygen species (ROS), and nitric oxide-mediated vasodilation, ultimately promoting growth factor expression and improved local microcirculation. However, PBM follows a biphasic dose–response relationship (Arndt–Schulz curve), whereby subtherapeutic doses produce negligible effects, while excessive irradiation may result in inhibitory or cytotoxic responses [118].

Studies on NIR (e.g., 808 nm) PBM for MRONJ primarily use a power density of 480 mW/cm^2^, an energy fluence of 6 J/cm^2^ per session (16 s exposure), and eight sessions over 4 weeks (total 48 J/cm^2^). This improved bone density, reduced dead bones, and enhanced epithelial regeneration in rat models compared to controls [119]. General PBM reviews recommend NIR fluences of 3–10 J/cm^2^ for stimulatory effects on tissues like bone, with power densities under 100–250 mW/cm^2^ to avoid inhibition, though higher values (up to 480 mW/cm^2^) succeeded in MRONJ models. Lack of guidelines stems from heterogeneous protocols; combined 660/808 nm shows promise, but human trials and scaffold-specific standardization are needed. For MRONJ stages (AAOMS classification), conservative PBM aids early stages, but parameters remain unoptimized [119,120,121].

Also, NIR-light-based treatments combined with photoadsorbers have demonstrated proven efficacy in bone cancer therapies, where photothermal and photodynamic mechanisms effectively ablate bone cancer cells while simultaneously supporting tissue regeneration, for example, through bifunctional nanomaterial-based scaffolds that combine anticancer activity with enhanced osteogenesis [122].

Over the years, PDT use has expanded beyond oncology to address complex conditions such as osteoradionecrosis (ORN) and MRONJ, where it leverages its potent antimicrobial and biomodulatory effects to promote healing in compromised tissues [123]. This therapy stands out for its precision, as it selectively targets pathological cells while minimizing damage to surrounding healthy structures, making it particularly suitable for patients who have undergone radiation or are on anti-resorptive medications, where traditional surgical interventions may pose higher risks.

One of PDT’s key strengths is its efficacy against antibiotic-resistant bacteria, such as *Staphylococcus aureus* and Actinomyces species, which are commonly implicated in secondary infections complicating ORN and ONJ [124]. By generating ROS that disrupt bacterial cell membranes and DNA without relying on traditional antimicrobial pathways, PDT circumvents resistance mechanisms that plague conventional antibiotics. To optimize outcomes and prevent the emergence of further resistance, PDT is frequently combined with empirical antibiotics, such as amoxicillin–clavulanate or metronidazole, providing a multifaceted approach to infection control [123].

So far, few studies have reported the use of PDT or the concomitant use of PDT and PBM in MRONJ treatment in humans [123].

In one of these studies, antimicrobial PDT has been applied immediately after tooth extraction using methylene blue as the photoadsorber, and irradiation was repeated weekly until total tissue repair. Subsequently, PBM therapy was applied until remission, which occurred for 16 out of 18 patients [125].

In addition to the infection limitation, PDT is known to modulate the immune system [126,127]. Indeed, in an animal model of osteonecrosis, the combination of PDT and PBM attenuated the severity of lesions and the inflammatory process due to a reduction in macrophages, T lymphocytes, and cytokines that stimulated the activity of these cells [128].

The most common photosensitizer is methylene blue (MB) dye, followed by phenothiazine chloride (PC), thiazine derivate (TD), povidone iodine (PI), and toluidine blue (TBO) [123]. Each PS is selected based on their affinity for microbial targets and compatibility with laser systems [129]. Also, the choice of PS and light parameters (e.g., power density, energy fluence, and exposure time) is tailored to the lesion’s stage and site, often involving multiple sessions to achieve complete epithelialization.

#### 6.3.1. NIR-Mediated Effects Using Graphene as Photosensitizer

Since MRONJ is a complex multifactorial disease involving impaired bone remodeling, inhibited angiogenesis, inflammation, infection, and immune dysfunction, on one hand, the intrinsic properties of graphene-based material would stimulate bone regeneration; on the other hand, light would modulate immunological responses. Graphene-based materials may enhance the biological effects of PBM and PDT not only by acting as passive photothermal agents but also through active optoelectronic interactions with incident light. Owing to their broad-spectrum optical absorption, high photoconductivity, and large specific surface area (up to 2600 m^2^/g), graphene derivatives can facilitate electron–hole separation under NIR irradiation, thereby promoting localized charge transfer reactions at the material–tissue interface. This process may increase the efficiency of ROS generation during PDT through improved photosensitizer excitation and singlet oxygen production, while simultaneously enabling photothermal conversion with minimal light scattering due to the planar sp^2^ carbon lattice. Under NIR stimulation (e.g., 808 nm), graphene’s plasmonic effects may further convert photon energy into localized heat with reported efficiencies of 58–67%, resulting in membrane permeabilization and enhanced light penetration within scaffold microenvironments.

In addition, graphene’s high thermal conductivity (~5000 W/mK) allows spatial confinement and uniform dissipation of photothermally generated heat, potentially amplifying mitochondrial cytochrome c oxidase activation during low-fluence PBM while avoiding bulk tissue overheating. Through its photoconductive properties, graphene may enable more efficient energy transfer from incident light to intracellular electron transport pathways, thereby supporting mitochondrial activation, ATP production, and the upregulation of pro-regenerative growth factors. Moreover, the high specific surface area of graphene enables π–π stacking interactions with biomolecules and facilitates oxygen transport within hypoxic environments, promoting pro-angiogenic signaling such as VEGF expression in stem cell niches. Collectively, these combined optoelectronic and photothermal effects suggest that graphene may function as a photoactive mediator capable of modulating both ROS-dependent antibacterial responses and ROS-independent mitochondrial signaling pathways in a fluence-dependent manner, ultimately synergizing PBM/PDT-driven tissue regeneration and angiogenesis [130,131,132,133].

For MRONJ, few examples have been reported in recent years. A 3D-printed polyetheretherketone (PEEK)–graphene scaffold coated with HA, referred to as PGH, was designed for remotely controlled bone regeneration through photothermal stimulation. When exposed to NIR light, the graphene phase generated mild hyperthermia around 42 °C, which, in turn, accelerated the release of Ca^2+^ and PO_4_^3−^ ions from the HA layer. The combined effect of localized heating and enhanced ion release significantly improved osteogenic activity and bone repair both in vitro and in vivo. Mechanistic studies revealed that these benefits were linked to the upregulation of HSP70, which triggered selective activation of the MAPK/ERK pathway to drive osteogenic differentiation. Animal models further demonstrated that PGH scaffolds under NIR irradiation produced markedly greater bone regeneration compared with non-irradiated groups [134].

Importantly, because both cells and bacteria are sensitive to surface micropatterns, a strategy combining such patterns with NIR stimulation was developed through a micropatterned graphene oxide nanocomposite on titanium implants (M-NTO/GO) for sequential infection control and osteogenic enhancement. The M-NTO/GO surface displayed a defined micropatterned nanostructure and showed responsiveness to NIR irradiation. When exposed to NIR light, M-NTO/GO exhibited strong antibacterial activity, reaching inhibition rates of 96.9% against *E. coli* and 98.6% against *S. aureus*. In the absence of irradiation, the micropatterned topography promoted guided cell growth, improved adhesion and spreading, and supported osteogenic differentiation. Overall, these results highlight the successful design of a multifunctional micropatterned nanocomposite implant capable of sequentially regulating antibacterial effects and bone-forming activity [135].

A facile self-assembly method was developed to fabricate a three-dimensional hydrogel scaffold composed of nanohydroxyapatite and reduced graphene oxide (nHA-rGO). The scaffold formed spontaneously during stirring and was stabilized by lyophilization and thermal reduction. When implanted at tumor sites and exposed to NIR (808 nm) irradiation, the scaffold rapidly increased local temperature to 60 °C within 4 min, effectively reducing tumor size and preventing further growth through photothermal therapy. In parallel, the scaffold supported adhesion, proliferation, and osteogenic differentiation of rat bone marrow stem cells in vitro and promoted bone regeneration in rat cranial defect models [136].

In addition, NIR-triggered controlled release systems, such as nanomaterials loaded with SDF-1α—a chemoattractant for EPCs—can further support cellular recruitment and the development of new vasculature within bone defects [122].

Copper nanoparticles and reduced graphene oxide have also been integrated into polylactic acid fibers (PLLA) through electrospinning and self-assembly, creating a composite material responsive to NIR light. Upon 808 nm irradiation, the system generated localized heating and ROS, achieving efficient photothermal and photodynamic antibacterial activity against *E. coli* and *S. aureus*. The incorporation of Dexamethasone Sodium Phosphate enabled controlled drug release, enhancing osteogenic differentiation, while copper ions promoted angiogenesis, both without inducing cytotoxicity and stimulating bone regeneration and vascularization [137].

Also, a recent study developed a TiO_2_/graphene metastructure on titanium implants through a combination of hydrothermal treatment and plasma-enhanced chemical vapor deposition, creating a surface with strong NIR absorption and high antibiotic loading capacity. Doxycycline was successfully adsorbed onto the graphene layer, and its release could be precisely controlled by NIR irradiation, enabling synergistic antibacterial action together with the photodynamic activity of TiO_2_. Both in vitro and in vivo experiments demonstrated that this system efficiently eradicated *E. coli* and *S. aureus* with short NIR exposure, while minimizing cytotoxicity compared to free antibiotics. Importantly, the TiO_2_/graphene surface promoted osteoblast proliferation, upregulated osteogenic markers such as OPN, BSP, and OCN, and enhanced new bone formation and bone–implant contact in rat models. Overall, this multifunctional coating provides a promising strategy for titanium implants, combining light-triggered antibiotic delivery, photodynamic antibacterial effects, and improved osseointegration [138].

#### 6.3.2. Time-Dependent Therapeutic Window for Graphene/NIR-Assisted Regeneration in MRONJ

Given the temporally evolving pathophysiology of post-extraction healing in MRONJ—characterized by an early infection-driven inflammatory phase followed by impaired angiogenesis and delayed osteogenic remodeling—the potential therapeutic effects of graphene-based scaffolds combined with NIR-mediated phototherapy should be interpreted within a stage-dependent biological framework. In the acute phase, the key pathological challenge is represented by persistent bacterial colonization and M1 macrophage-driven inflammation occurring in a hypoxic microenvironment with reduced vascular clearance. In this context, the photothermal and photodynamic properties of graphene under NIR irradiation may enable localized antibacterial action through ROS generation and membrane disruption, as supported by recent NIR-responsive graphene-based scaffold studies demonstrating inhibition rates exceeding 95% against common oral pathogens. However, excessive or prolonged irradiation in an already inflamed environment may increase oxidative stress and compromise endothelial viability, thereby narrowing the effective therapeutic window.

With regard to the anti-inflammatory switch required to promote tissue regeneration and neovascularization, the possibility of precisely modulating light parameters to transition from PDT to PBM opens additional therapeutic perspectives. In this context, graphene-based materials may be advantageously combined with nanoparticles or bioactive ions capable of exerting dual antimicrobial and pro-regenerative action.

Among these, copper ions have demonstrated broad-spectrum antibacterial activity through multiple mechanisms, including disruption of bacterial cell membranes, generation of ROS, and interference with essential microbial enzymes and DNA replication. When incorporated into NIR-responsive platforms, such as MXene-based photothermal hydrogels, copper ions can be released upon light stimulation, contributing not only to antibacterial effects but also to ROS scavenging and the promotion of angiogenesis in infected environments [139].

A similar multifunctional approach has been reported for bovine serum albumin–iridium oxide nanoclusters (BSA-IrOx NCs), which enable light-regulated ROS modulation under near-infrared (NIR) irradiation. Upon laser exposure, these nanoclusters enhance photodynamic activity, facilitating biofilm disruption and bacterial killing. Conversely, in the absence of irradiation, their intrinsic antioxidant enzyme-like properties contribute to inflammation control and tissue repair through ROS clearance. Multi-omics analyses further suggest that these nanoclusters inhibit bacterial nitric oxide synthase, thereby impairing bacterial growth and biofilm formation. In parallel, they have been shown to support tissue recovery by reinforcing intercellular junctions and reducing mitochondrial damage in fibroblast models [140].

As healing progresses into the transition phase, MRONJ lesions are predominantly characterized by impaired angiogenesis associated with suppressed VEGF signaling and endothelial dysfunction.

In the context of chronic pathologies characterized by impaired vascularization, such as MRONJ, the local administration of pro-angiogenic growth factors in a soluble or bolus form has consistently shown limited therapeutic efficacy due to their short biological half-life, rapid diffusion from the implantation site, and proteolytic degradation within inflamed tissues. In contrast, scaffold-mediated delivery systems have been widely demonstrated to improve therapeutic angiogenesis by providing spatially and temporally controlled release of bioactive molecules.

Under physiological conditions, most angiogenic growth factors are not freely soluble but are retained within the extracellular matrix through interactions with heparan sulfate proteoglycans and fibronectin, forming local concentration gradients that regulate endothelial migration and vessel maturation. Biomaterial-based scaffolds aim to replicate this native storage function by incorporating growth factors within hydrogel or porous matrices capable of modulating their release kinetics through matrix degradation, electrostatic interactions, or affinity binding [141].

Several general strategies have been developed to achieve sustained angiogenic signaling from scaffolds. These include covalent immobilization of growth factors to polymeric backbones, incorporation into affinity-binding matrices such as gelatin or alginate-sulfate systems, and encapsulation within nanoparticle or hydrogel compartments embedded in the scaffold architecture. These approaches enable the gradual liberation of bioactive molecules as a function of scaffold biodegradation or local environmental conditions, thereby significantly reducing the initial burst release typically associated with direct adsorption or injection [142].

Importantly, experimental studies have demonstrated that the degradation-dependent release of VEGF from collagen hydrogels results in prolonged retention of biologically active protein in vivo and induces significantly greater angiogenesis compared with VEGF administered in solution form. In such systems, slower scaffold degradation correlates with extended growth factor availability and sustained neovascularization over time [143].

Alternatively, next-generation engineered living materials may overcome the intrinsic limitations of passive scaffold-mediated delivery by enabling the in situ synthesis of therapeutic molecules in a dynamically regulated manner. Living materials incorporate metabolically engineered probiotic microorganisms encapsulated within hydrogel matrices capable of continuously producing bioactive compounds directly at the target site [144,145]. In optogenetically controlled systems, protein synthesis can be remotely activated by external stimuli such as light, thereby enabling spatially confined and temporally tunable release profiles that are not achievable with traditional encapsulation technologies, with secretion levels adjustable as a function of light dose and stimulation cycles. As an example, NIR-responsive engineered living materials have been shown to produce and secrete VEGF-mimetic peptides in a controllable manner when encapsulated within alginate core–shell hydrogels, ensuring both bacterial biocontainment and sustained diffusion of the therapeutic payload into the surrounding environment [144].

In the later proliferative phase, the principal biological limitation shifts toward uncoupled osteogenesis and defective extracellular matrix deposition resulting from persistent osteoclast inhibition. Under these conditions, delayed low-fluence NIR exposure may induce mild photothermal effects (~42 °C) capable of activating HSP70-mediated MAPK/ERK signaling pathways, thereby promoting mesenchymal stem cell osteogenic differentiation and mineralized matrix formation. Preclinical evidence from NIR-activated graphene composite scaffolds indicates that such sequential stimulation may enhance ion release kinetics and support bone regeneration in vivo.

In parallel, GO is known to undergo progressive physicochemical modifications in biological environments, where the adsorption of proteins and the presence of ionic species may promote a time-dependent reduction in GO toward more conductive rGO-like states [146].

This process may be further accelerated by photothermal stimulation, ultimately modulating the surface chemistry and promoting the biological accumulation of carbonate and phosphate ions that sustain the formation of a biomimetic mineralized layer. Notably, rGO has been shown to maintain enhanced osteogenic potential following conditioning layer formation, supporting mineralized matrix deposition and mesenchymal stromal cell proliferation [75,147]. Altogether, these observations suggest that NIR-induced thermal stimulation may not only activate cellular pathways but also dynamically tune the osteoinductive properties of graphene-based scaffolds over time through progressive surface reduction and hydroxyapatite nucleation.

**Figure 6:**
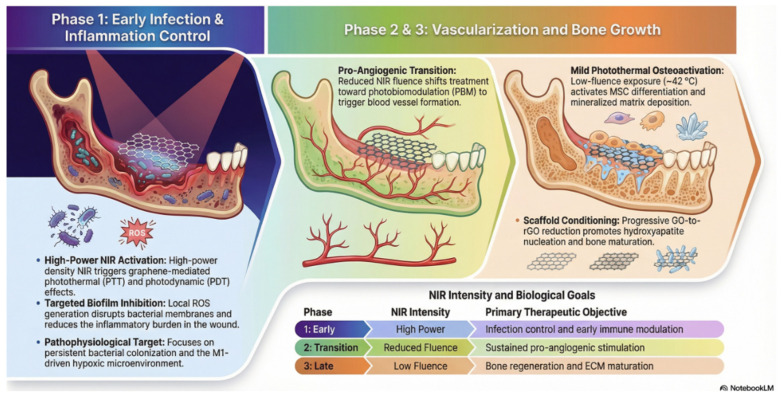
The graphene-mediated sequential strategy for MRONJ treatment, created with NotebookLM.

Taken together, these observations support a conceptual model in which multifunctional graphene-enabled implants could be engineered for sequential regulation of the MRONJ healing cascade, providing early antimicrobial photothermal activity followed by sustained pro-angiogenic and osteogenic guidance through scaffold-mediated signaling, as shown in Figure 6. Future research should therefore focus on validating stage-adapted, NIR-responsive scaffold systems in MRONJ-specific in vitro and in vivo models.

### 6.4. Limitations and Future Directions for Graphene-Based Materials Application

Despite promising regenerative and antimicrobial outcomes, translation of graphene-based scaffolds to MRONJ care might be currently limited by (i) cytotoxicity studies and (ii) the narrow and context-dependent MRONJ therapeutic window.

Graphene layer-induced cytotoxicity is highly dependent on physicochemical parameters such as layer number [148], lateral size [149], and oxidation state [150], with smaller single-layer graphene oxide (SLGO) typically exhibiting greater biological reactivity and cellular internalization, thereby exerting stronger inhibitory effects on cell proliferation than multi-layer counterparts, although few-layer graphene (FLG) has shown enhanced toxicity in selected immune cell populations under specific conditions [148]. In parallel, the oxidation state plays a critical role, as rGO is often more cytotoxic than GO due to decreased oxygen-containing functional groups that increase hydrophobic interactions with cellular membranes, ultimately promoting higher ROS generation and reduced cell viability, while lateral size further modulates these effects, since smaller GO sheets (e.g., ~20 nm) generally display higher dose-dependent toxicity than larger sheets (e.g., ~100 nm) owing to their greater surface area and more efficient cellular uptake. Importantly, surface functionalization strategies such as PEGylation can mitigate these adverse effects by improving colloidal stability and biocompatibility, whereas the dominant mechanism of toxicity remains dose-dependent oxidative stress associated with mitochondrial membrane potential disruption and apoptosis induction, with significant biological effects frequently emerging only after prolonged exposure [151]. It must be noted that the toxicity of commercially available graphene materials may vary substantially due to residual impurities or synthesis-related contaminants, complicating the attribution of observed cytotoxic responses to graphene structure alone [152]. This problem is amplified by batch-to-batch variability: Different synthesis routes (oxidation degree, lateral size, thickness, and residual metals) and post-functionalization (e.g., polydopamine, chitosan, PEGylation, and thermal reduction) can produce materials with non-equivalent biological responses, even when all are labeled “GO/rGO”. Standardized characterization and reporting (C/O ratio, flake size distribution, thickness, ζ-potential, endotoxin and residual catalyst/metal content, and stability/aggregation in relevant biological media) should be treated as a prerequisite for comparing studies and for defining a clinically meaningful dose window. Recent bone-regenerative GO literature highlights both the need for reproducible, scalable synthesis and the translational concern created by synthesis-dependent variability [130,153].

In the inflamed, hypoxic, and infection-prone jaw environment—further perturbed by anti-resorptive/anti-angiogenic therapies—small changes in the local effective dose can shift GO from pro-regenerative signaling to oxidative stress, endothelial dysfunction, and impaired angiogenesis. This is consistent with the strong dose-dependence reported for GO-containing hydrogels (0.1–0.5 wt.% pro-angiogenic/pro-healing vs. 1.0 wt.% cytotoxic/oxidative) [105] and with surface-chemistry effects on EPC viability and VEGFR2 signaling, where soluble GO downregulated VEGFR2 while thermally reduced rGO (particularly rGO30) mitigated toxicity and preserved or even enhanced VEGFR2 expression [104]. Our group has also demonstrated that the exposure to GO-loaded PLGA scaffolds resulted in limited direct cytotoxicity on unstimulated PBMCs, with no significant changes in cell viability, apoptosis, or necrosis observed across increasing GO concentrations, and no detectable increase in scaffold-derived ROS or superoxide release in the culture medium. However, under immune activation conditions, higher GO loadings (particularly 2–5%) induced a modest but measurable decline in PBMC viability and significantly impaired T-cell proliferative responses in a concentration-dependent manner, alongside reduced expression of activation markers such as CD25 and altered CD4^+^ T-cell differentiation profiles, including decreased Th1 polarization and a relative shift toward Th2 subsets. These findings suggest that, in scaffold-embedded configurations, GO does not primarily exert overt cytotoxic effects but rather modulates immune cell activation, proliferation, and differentiation in a dose-dependent manner, highlighting a more subtle immunomodulatory impact at higher concentrations [127]. However, when GO is incorporated within three-dimensional scaffolds, cytotoxicity appears to be governed less by intrinsic layer number and more by surface exposure, interfacial chemistry, and release kinetics at the biomaterial–tissue interface. In this context, the local release of graphene derivatives may still induce cytotoxic effects that are further modulated by the rapid formation of a so-called protein corona—a dynamic layer of adsorbed host proteins that spontaneously coats nanomaterial surfaces upon contact with biological fluids—thereby redefining their biological identity, cellular interactions, and effective bioavailability in a patient-specific manner depending on the inflammatory milieu and circulating protein profile. Future work should therefore quantify the concentration actually experienced at the tissue interface (accounting for release kinetics, adsorption to proteins/ECM, protein corona formation, and sedimentation) rather than only the nominal loading in the scaffold.

To test the MRONJ-specific hypothesis in a rigorous way, key in vitro experiments should be performed under “MRONJ-like” stressors (zoledronate or denosumab exposure, pro-inflammatory cytokines/LPS, low pH, hypoxia, and polymicrobial biofilm challenge) using relevant cell types in monoculture and co-culture: (i) oral keratinocytes and gingival fibroblasts for mucosal closure and barrier integrity; (ii) macrophages/monocytes to map inflammasome activation and M1↔M2 transitions; (iii) osteoclast precursors to quantify rescue or further suppression of osteoclastogenesis; (iv) MSCs/DPSCs/osteoblasts for osteogenic differentiation; and (v) endothelial cells/EPCs for angiogenesis assays (tube formation/sprouting, VEGFR2/VEGF signaling). These studies should include full dose–response curves with clinically plausible exposure metrics (mass and surface area per mL and per cm^2^), direct measurements of ROS/mitochondrial stress, and stability/aggregation readouts in the same media used for biology. In vivo, the most informative designs would integrate GO-based implants into validated MRONJ models (e.g., rodent tooth-extraction models under zoledronate ± corticosteroids or denosumab) and include quantitative endpoints beyond bone fill: incidence/duration of bone exposure, microbial burden and biofilm, vascular density, and immune infiltrates, and systemic biodistribution/clearance of possible released GO/rGO. If photothermal therapy is provided, multifunctional constructs should be evaluated as sequential systems: a first photothermal cycle to reduce bacterial load, followed by a mechanically stable, pro-angiogenic/osteogenic phase with controlled release of growth factors or immunomodulators—while ensuring that repeated NIR irradiation does not widen the toxicity window in already inflamed tissues.

## 7. Conclusions

In the evolving landscape of surgical and dental therapeutics, graphene-based materials represent a promising and innovative frontier in the management of MRONJ. Their multifunctional properties, combining exceptional mechanical strength, biocompatibility, modulation of osteogenic and immune cellular responses, and integration with NIR-mediated phototherapies open new and tangible opportunities to enhance current therapeutic strategies. Preclinical studies indicate that graphene-coated implants support osteogenic differentiation of stem cells, reduce bacterial colonization, and provide mechanical resilience through tailored surface energy and coating durability. Reduced graphene oxide in composite scaffolds has been shown to foster new bone formation and mineralized tissue deposition more effectively than control materials [17].

The ability to link graphene-based scaffolds with the controlled release of pro-angiogenic factors and antimicrobial agents, together with photothermal and photodynamic effects, introduces a theragnostic paradigm capable of addressing the complex biological causes of MRONJ in an integrated manner.

Clinically, implementing these materials could lead to more effective surgical and conservative protocols, significantly reducing necrosis risks and promoting bone and vascular tissue regeneration in compromised sites. However, translation into routine clinical practice requires rigorous long-term safety validation, functional stability, production standardization, and cost-effectiveness assessment.

The multifaceted pathogenesis of MRONJ suggests that a single treatment approach may be insufficient, whereas the unique properties of graphene—such as osteogenic promotion, antibacterial effects, and compatibility with photodynamic activation—could synergize with NIR PDT’s targeted antimicrobial and regenerative capabilities to provide a more effective therapeutic strategy. This integrative concept aligns with the emerging paradigm of theragenerative and the repair materials, which combine therapy with regeneration or repair, respectively [138,154]. Key future perspectives include:

- Development of patient-specific 3D-printed graphene-functionalized scaffolds tailored to bone defect geometries, capable of dynamically modulating the local microenvironment upon external stimuli such as NIR light to enable targeted drug and growth factor delivery.
- Integration of multimodal release systems combining microRNAs, growth factors, and antimicrobials that simultaneously regulate osteogenesis, angiogenesis, and immune responses, paving the way for highly personalized, minimally invasive therapies.
- Exploitation of graphene’s photothermal and photodynamic properties to design combined therapies that effectively eradicate microbial biofilms while stimulating tissue regeneration, overcoming limitations in depth and specificity inherent to conventional treatments.
- Further elucidation of graphene-mediated immunomodulatory mechanisms, particularly macrophage polarization balance (M1 vs. M2), which is crucial for controlling chronic inflammation and facilitating bone healing, to refine immunomodulatory biomaterial designs.

Ultimately, advancing collaboration between material scientists, cell biologists, clinicians, and photomedicine experts will be essential to accelerate clinical translation and broaden access to these technologies, heralding a new era of personalized, effective, and safe treatment options for patients affected by MRONJ.

## Data Availability

No new data were created or analyzed in this study.

## Supplementary Materials

The following supporting information can be downloaded at: https://www.mdpi.com/article/10.3390/pharmaceutics18030335/s1, File S1: PRISMA 2020 Checklist.
